# Supplementary material for: Cost savings in male circumcision post-operative care using two-way text-based follow-up in rural and urban South Africa
Source: PLoS One. 2023 Nov 16;18(11):e0294449. doi: 10.1371/journal.pone.0294449 (PMC10653449; doi:10.1371/journal.pone.0294449)
Supplement: S1 File — Tool used for 2WT time-in-motion client follow-up data collection. (PDF) [file pone.0294449.s001.pdf]

2WT Costing

Client followup

District

- ○ Bojanala
- ○ Ekurhuleni

Who is completing this form?

- ○ [redacted]
- ○ [redacted]
- ○ [redacted]

Date today of follow-up

Time started travelling to client

Car odometer reading at start of journey to client

Time arrived at client

Car odometer reading at arrival at client

Please click here for GIS details

Time spent with client (in minutes)

Client Age

Client follow-up visit type

- ○ Day 2
- ○ Day 7
- ○ Day 14
- ○ LTFU Tracing effort
- ○ AE review

Please add any information here about this visit
