# Supplementary material for: Cost savings in male circumcision post-operative care using two-way text-based follow-up in rural and urban South Africa
Source: PLoS One. 2023 Nov 16;18(11):e0294449. doi: 10.1371/journal.pone.0294449 (PMC10653449; doi:10.1371/journal.pone.0294449)
Supplement: Attachment — Submitted filename: Reviewer Report -- 2wT VMMC.docx [file pone.0294449.s003.docx]

Reviewer Report

Cost savings in male circumcision post-operative care continuum in rural and urban

South Africa: Evidence on the importance of initial counselling and daily SMS

**Summary**

1. Severe adverse events (AEs) are relatively rare after VMMC in South Africa and elsewhere. As a result, requiring men to attend multiple post-op visits likely wastes men’s time, wastes public resources, and probably adds to congestion at already crowded clinics. Rather than requiring men to return to clinics, implementing two-way text messaging (2wT) during the 14-day post-op period yielded good results in Zimbabwe, and it seems the same team implemented a similar study in South Africa (Feldacker PI). While the current costing manuscript provides a reference 15 for the main study, I could not find this reference online to review the primary results of the study, which is useful for understanding the evaluation of costs of services in each study arm.

2. As provided by the authors (line 87), “The objective this study was to estimate the costs associated with 2wT telehealth follow-up as compared to routine in-person VMMC follow-up in rural and urban South Africa.” And on line 162, costs were to be estimated using an “activity-based costing approach (with 4 main activities: counseling, sms follow up, physical follow up, and tracing).

**Main comments.**

3. While the objective and costing approach are both logical, my basic comment on this analysis is that costing approach presented here is not logically developed or presented. I’ll explain more below.

4. In principle, the evaluation of costs of services provided for post-op care for patients in each study arm requires information on the quantity of resources provided to each patient (e.g., services at clinic visits; 2wT services, etc.) and a unit cost for each service. With 537 clients in standard care and 547 in the intervention group, a data set with 1084 observations containing the list of resources included in the costing analysis along with the quantities should exist. This is often call the resource use matrix (see, e.g. <https://www.heroza.org/researchtools/the-healthcare-cost-and-outcomes-model-hcom/> ). This dataset can then also include any primary or secondary study outcomes as relevant for a study. For documentation purposes, this dataset (sometimes called a resource use matrix) should be provided as a supplemental file with this paper (or the dataset could be put in a data repository; deidentify as needed but the basic resource variables required for costing do not identify clients). This also implies that the data availability statement is not adequate. All relevant data are not provided within the manuscript.

I would highly recommend that you follow this approach for your costing analysis as explained by the HERO costing model. You can look at the Scott et al. article for an example of applying this approach: <https://bmcpublichealth.biomedcentral.com/articles/10.1186/1471-2458-14-296>

Note: This study has separate unit costs for each site in the study (for some resources).

A similar type of analysis for clinical trial is used in this study:

<https://www.thelancet.com/journals/langlo/article/PIIS2214-109X(21)00039-5/fulltext>

Look at supplemental appendix 2 for costing details.

One you revise your analysis and rewrite your manuscript (basically a new paper), you will then have a logically presented cost analysis for your topic.

**The comments below are just some extra comments or information related to how you can revise your analysis and presentation.**

5. As noted on line 164, there were four activity categories in costing: counselling on the day of male circumcision, SMS follow ups, physical follow-up visits, and tracing. With activity-based costing, I’d expected to see a description of how a unit cost for each activity would be created (and then combined with the resource use matrix) to estimate a cost for each activity and then a total cost of post-op follow up for each patients (the set of unit costs can also be provided with the resource use matrix). With this information, standard summary statistics can be provided (mean costs per client by study arm, median/IQR, etc.) and then you can estimate the mean difference between study arms (with 95% CI, etc.).

6. I’m not sure the cost of AE treatment should be considered a cost for this analysis. In Zimbabwe, the 2wT increased slightly the identification of AEs (as the authors note due “improved AE ascertainment”). If 2wT serves as a proxy for active surveillance, identifying AEs is actually a benefit of the intervention. <https://journals.lww.com/jaids/Fulltext/2020/01010/Reducing_Provider_Workload_While_Preserving.3.aspx>). It seems this is basically a type of information bias, in that AEs are likely identified better in one study arm.

7. Line 94 – since you are estimating costs based on a two-arm individually randomized clinical trials, I suggest just using Study Arms or Study Groups

8. Line 139 – How were data collection tools piloted? These tools/files can also be provided as a supplemental file or with the data repository mentioned above.

9. Line 148 – Line 149: While not a big deal, it would help to be more specific on the assumptions used to estimate a nurse wage per hour (I assume used with time information to get a time cost for various services). First, regarding wage per hour, the authors need to confirm that they obtained the total cost to employer. In addition, I assume nurses have days for holidays, annual leave, perhaps other types. These total days off need to be subtracted from some maximum possible to come up with a wage per hour working.

10. Line 154-160 – These hypotheses are rather odd for this costing paper. For example, this costing paper is designed to estimate the cost of post-op care by study arm. Once the costs by study are presented (e.g. mean(SD), median(IQR)), it is then possible to estimate a mean difference with a 95% CI. The second topic is simply the same analysis stratified by urban rural (so provide the same results stratified by urban/rural). I’m not sure why Hyp 3 is included. As presented in Table 1, the 2wT arm does not require post-op visits.

11. Line 165 – Table 2 provides some sort of summary results for various stuff, but I would not consider them “parameters”. I have no idea why you mention 50%+- uncertainty intervals here either.

12. Line 181 – I now see what the authors are trying to do. I’ll break out the SMS cost in detail as described in the paragraph above line 181.

SMS cost =	set-up cost +

SMS unit cost * average number SMS per client +

(1/60)*nursewage*average number of SMS per client

I think it would be much better to use the actual patient level data along with the unit cost to first estimate the SMS cost per patient. With 500+ observations in each study arm (but SMS only in one arm), you have 500+ SMS cost numbers (which later you can summarize, etc.).

The resource use matrix would have the follow quantity resources:

X1 = Set up SMS for client (0 for standard care, 1 for 2wT arm)

X2 = Number of SMS to client (0 for standard care, looks like 13 if exactly one each day, but like more or less for some clients).

A unit cost for each resource is then:

C1 = cost to set up SMS

C2 = cost per SMS = (SMS unit cost + (1/60)*wage)

So for each client, SMS cost = C1*X1 + C2*X2

In Line 174, this same process can be used for counseling time, unless you do not have counselling time captured per client. It’s OK if you just have a counseling time by study arm (seems like 2wT arm has longer post counseling).

13. Line 191 – Here again, you can have the number of rural visits and number of urban visits per patient from your data, and then it looks like you have one “unit cost” for a rural visit.

14. I suspect you have an Excel file that includes all the calculations you used to estimate a transport fuel cost and transport time cost, etc. It would be good to add that file to the study data repository.

15. In line 194, do you have a fuel cost per client? Or are these based on some assumed averages? If you do, then you can have a fuel cost per client per visit that is specific to each client. In this case, you’d have km roundtrip to client as a quantity in the resource use matrix. For each client, the number of rural trips and urban trips would be extra quantity variables in the resource use matrix. Petrol price would then be an extra unit cost to add into the analysis and fuel economy would then be an extra parameter used to estimate the fuel cost per client (and similar logic for transport time, review time, etc.

16. It seems some asset value per trip (for the vehicle) is needed that included the asset value plus other costs (annual road taxes, license, maintenance, insurance, etc.)?

17. Line 203 -- for phone tracing, you have the number of tracing attempts per client, so use that directly (e.g., # unsuccessful attempts over follow up period, # successful). Then you have a unit cost for each type of event.

18. Line 211 – same issue here for tracing.

19. Line 221 – It is not clear what the authors did for the analysis presented in Table 3.

20. Line 225 – The purpose of Table 2 and the information presented in Table 2 is unclear. You have two study arms. I highly recommend you follow your earlier stated objective (estimate costs in each study arm using activity-based costing and I’d add the actual patient level data that you have.

Actually Table 4 comes close to what you want to report. Once you do the client level cost analysis, you can present the resource quantities per client, the unit costs that go with each resource, and the costs by resource. You can report for all means(SD) and median (IQR),, etc, and then estimate the mean differences (with 95% CI). A separate table can then report the same information stratified by urban/rural. By the way, is this urban/rural clients or where the clinics are located?

21. I’m not going to add a lot of additional comments on the rest of the paper (e.g., issue in line 317 – Table shows cost of post op care, not per visit I think), given that the methods and presentation needs to be fully revised. With the revised paper, the section on “other scenarios” doesn’t really make sense. Check line 428.
