# Supplementary material for: Cost savings in male circumcision post-operative care using two-way text-based follow-up in rural and urban South Africa
Source: PLoS One. 2023 Nov 16;18(11):e0294449. doi: 10.1371/journal.pone.0294449 (PMC10653449; doi:10.1371/journal.pone.0294449)
Supplement: Attachment — Submitted filename: Response to Reviewers 6.30.2023.docx [file pone.0294449.s004.docx]

We thank the editor and reviewers for reviewing this paper and providing constructive comments and suggestions. Point-to-point response to each comment is provided here (underlined), and corresponding changes in the manuscript are tracked.

Best,

Yanfang, Rachel, Caryl and co-authors

PONE-D-23-01844

**Part 1: Journal Requirements:**

When submitting your revision, we need you to address these additional requirements.

1. Please ensure that your manuscript meets PLOS ONE's style requirements, including those for file naming. The PLOS ONE style templates can be found at 
[https://journals.plos.org/plosone/s/file?id=wjVg/PLOSOne_formatting_sample_main_body.pdf](https://urldefense.com/v3/__https:/eur01.safelinks.protection.outlook.com/?url=https*3A*2F*2Furldefense.com*2Fv3*2F__https*3A*2F*2Feur01.safelinks.protection.outlook.com*2F*3Furl*3Dhttps*3A*2F*2Furldefense.com*2Fv3*2F__https*3A*2Fjournals.plos.org*2Fplosone*2Fs*2Ffile*3Fid*3DwjVg*PLOSOne_formatting_sample_main_body.pdf__*3BLw!!K-Hz7m0Vt54!iInB6z4ybzPWlCG2gI2YNHJIr3qlZwTOL_v1dAqDfBVTAru_5vyCkkuDN28LBQJLMQX_bui_Hg5RJA*24*26data*3D05*7C01*7CGSetswe*40auruminstitute.org*7Cc639dd7a75484c4d1f4808db1c063740*7C706824277a664bae9f42f702a8ba8b2a*7C0*7C0*7C638134585501549353*7CUnknown*7CTWFpbGZsb3d8eyJWIjoiMC4wLjAwMDAiLCJQIjoiV2luMzIiLCJBTiI6Ik1haWwiLCJXVCI6Mn0*3D*7C2000*7C*7C*7C*26sdata*3DAMCF54*2F4WZu1c5sMjlZCJ5ijE1HrS93BkzDM3GU*2FyBI*3D*26reserved*3D0__*3BJSUlJSUlJSUlJSUlKiUlJSUlJSUlJSUlJSUlJSUlJSUl!!K-Hz7m0Vt54!igS3sJLUCpHGFDsPHZwmkI7hh1g7teQjp67FwKod5uZPLk3LnSSqek-q6KWJ4rDZk7PvNhdmMhOjx38evE8*24&data=05*7C01*7Crmukora*40auruminstitute.org*7Cf0dc02085582472f4c6908db1df7e6b6*7C706824277a664bae9f42f702a8ba8b2a*7C0*7C0*7C638136723204775393*7CUnknown*7CTWFpbGZsb3d8eyJWIjoiMC4wLjAwMDAiLCJQIjoiV2luMzIiLCJBTiI6Ik1haWwiLCJXVCI6Mn0*3D*7C3000*7C*7C*7C&sdata=Og*2Bt1RbQA3s6gbQXQaBKloW4ZNsRWjh6EdXnvgWmiic*3D&reserved=0__;JSUlJSUlJSUlJSUqKioqKioqKioqKioqKiolJSoqKioqKioqKioqKioqKiUlKioqJSUlJSUlJSUlJSUlJSUlJSUlJSUl!!K-Hz7m0Vt54!iJ0i0UU-hjYj9FMAXIBNliosAbEuCa07IQoTA4EGsCLHhE2hyklN6QLFPRkRatB-FDTU4RES0ohpcbOMwuo$) and 
[https://journals.plos.org/plosone/s/file?id=ba62/PLOSOne_formatting_sample_title_authors_affiliations.pdf](https://urldefense.com/v3/__https:/eur01.safelinks.protection.outlook.com/?url=https*3A*2F*2Furldefense.com*2Fv3*2F__https*3A*2F*2Feur01.safelinks.protection.outlook.com*2F*3Furl*3Dhttps*3A*2F*2Furldefense.com*2Fv3*2F__https*3A*2Fjournals.plos.org*2Fplosone*2Fs*2Ffile*3Fid*3Dba62*PLOSOne_formatting_sample_title_authors_affiliations.pdf__*3BLw!!K-Hz7m0Vt54!iInB6z4ybzPWlCG2gI2YNHJIr3qlZwTOL_v1dAqDfBVTAru_5vyCkkuDN28LBQJLMQX_buiWmtvaeQ*24*26data*3D05*7C01*7CGSetswe*40auruminstitute.org*7Cc639dd7a75484c4d1f4808db1c063740*7C706824277a664bae9f42f702a8ba8b2a*7C0*7C0*7C638134585501549353*7CUnknown*7CTWFpbGZsb3d8eyJWIjoiMC4wLjAwMDAiLCJQIjoiV2luMzIiLCJBTiI6Ik1haWwiLCJXVCI6Mn0*3D*7C2000*7C*7C*7C*26sdata*3DDnYxdnEp7dgoMkLrAsMSiEX3IhGUw42p7HppPSV3TjE*3D*26reserved*3D0__*3BJSUlJSUlJSUlJSUlKiUlJSUlJSUlJSUlJSUlJSUlJQ!!K-Hz7m0Vt54!igS3sJLUCpHGFDsPHZwmkI7hh1g7teQjp67FwKod5uZPLk3LnSSqek-q6KWJ4rDZk7PvNhdmMhOj0IgKH6A*24&data=05*7C01*7Crmukora*40auruminstitute.org*7Cf0dc02085582472f4c6908db1df7e6b6*7C706824277a664bae9f42f702a8ba8b2a*7C0*7C0*7C638136723204775393*7CUnknown*7CTWFpbGZsb3d8eyJWIjoiMC4wLjAwMDAiLCJQIjoiV2luMzIiLCJBTiI6Ik1haWwiLCJXVCI6Mn0*3D*7C3000*7C*7C*7C&sdata=1wi45vUUQposXaSYO5jO33E3*2Fq8NR682I3bj48j4N2w*3D&reserved=0__;JSUlJSUlJSUlJSUqKioqKioqKioqKioqKiolJSoqKioqKioqKioqKioqKiUlKiUlJSUlJSUlJSUlJSUlJSUlJSUlJQ!!K-Hz7m0Vt54!iJ0i0UU-hjYj9FMAXIBNliosAbEuCa07IQoTA4EGsCLHhE2hyklN6QLFPRkRatB-FDTU4RES0ohpB9gdBHc$)

Response: we have followed PLOS ONE’s style requirements and we updated file manes.

2. Please provide additional details regarding participant consent. In the ethics statement in the Methods and online submission information, please ensure that you have specified (1) whether consent was informed and (2) what type you obtained (for instance, written or verbal, and if verbal, how it was documented and witnessed). If your study included minors, state whether you obtained consent from parents or guardians. If the need for consent was waived by the ethics committee, please include this information.

If you are reporting a retrospective study of medical records or archived samples, please ensure that you have discussed whether all data were fully anonymized before you accessed them and/or whether the IRB or ethics committee waived the requirement for informed consent. If patients provided informed written consent to have data from their medical records used in research, please include this information.

Response: Only aggregate, de-identified patient data on client visits and adverse events from the RCT was used for costing analysis. Individual level data was not analyzed for this costing study. We added the following information in the ethics statement in the Methods section.

This Multiple Principal Investigators (MPI) study was approved by the Internal Review Boards of the University of Washington (Study 00009703, PI: Feldacker) and the University of the Witwatersrand, Human Research Ethics Committee (Ethics Reference No: 200204, PI: Setswe). All RCT participants provided written informed consent for use of individual-level data collected from both 2wT study specific sources and routine VMMC program data collection. All data obtained and utilized for the costing study was de-identified data from study databases and contained no identifying information. As part of the overall RCT IRB approvals, a waiver of consent was granted for healthcare worker observations from the time-motion study.

3. Thank you for stating in your Funding Statement: 
"Research reported in this publication was supported by the National Institute of Nursing Research (NINR) of the National Institutes of Health under award number 5R01NR019229, “Expanding and Scaling Two-way Texting to Reduce Unnecessary Follow-Up and Improve Adverse Event Identification Among Voluntary Medical Male Circumcision Clients in the Republic of South Africa.”

Please provide an amended statement that declares *all* the funding or sources of support (whether external or internal to your organization) received during this study, as detailed online in our guide for authors at [http://journals.plos.org/plosone/s/submit-now.](https://urldefense.com/v3/__https:/eur01.safelinks.protection.outlook.com/?url=https*3A*2F*2Furldefense.com*2Fv3*2F__https*3A*2F*2Feur01.safelinks.protection.outlook.com*2F*3Furl*3Dhttps*3A*2F*2Furldefense.com*2Fv3*2F__http*3A*2Fjournals.plos.org*2Fplosone*2Fs*2Fsubmit-now.__*3B!!K-Hz7m0Vt54!iInB6z4ybzPWlCG2gI2YNHJIr3qlZwTOL_v1dAqDfBVTAru_5vyCkkuDN28LBQJLMQX_bugU_2zh6w*24*26data*3D05*7C01*7CGSetswe*40auruminstitute.org*7Cc639dd7a75484c4d1f4808db1c063740*7C706824277a664bae9f42f702a8ba8b2a*7C0*7C0*7C638134585501549353*7CUnknown*7CTWFpbGZsb3d8eyJWIjoiMC4wLjAwMDAiLCJQIjoiV2luMzIiLCJBTiI6Ik1haWwiLCJXVCI6Mn0*3D*7C2000*7C*7C*7C*26sdata*3DXD4lL9E1tsmFEnDIO11nnPnH47CjNwOMB4OwQ0AYkd4*3D*26reserved*3D0__*3BJSUlJSUlJSUlJSUlJSUlJSUlJSUlJSUlJSUlJQ!!K-Hz7m0Vt54!igS3sJLUCpHGFDsPHZwmkI7hh1g7teQjp67FwKod5uZPLk3LnSSqek-q6KWJ4rDZk7PvNhdmMhOjo64nwjk*24&data=05*7C01*7Crmukora*40auruminstitute.org*7Cf0dc02085582472f4c6908db1df7e6b6*7C706824277a664bae9f42f702a8ba8b2a*7C0*7C0*7C638136723204775393*7CUnknown*7CTWFpbGZsb3d8eyJWIjoiMC4wLjAwMDAiLCJQIjoiV2luMzIiLCJBTiI6Ik1haWwiLCJXVCI6Mn0*3D*7C3000*7C*7C*7C&sdata=BTPv0CZHB1Sn7UcK9TRtRw18GqLCk2RsoKqiRo6tqFA*3D&reserved=0__;JSUlJSUlJSUlJSUqKioqKioqKioqKiolJSoqKioqKioqKioqKioqKiUlKiUlJSUlJSUlJSUlJSUlJSUlJSUl!!K-Hz7m0Vt54!iJ0i0UU-hjYj9FMAXIBNliosAbEuCa07IQoTA4EGsCLHhE2hyklN6QLFPRkRatB-FDTU4RES0ohpqaso2Hc$)  Please also include the statement “There was no additional external funding received for this study.” in your updated Funding Statement. 
Please include your amended Funding Statement within your cover letter. We will change the online submission form on your behalf.

Response: We have amended the funding statement in the manuscript and included it within the cover letter.

4. Thank you for stating the following financial disclosure: 
"Research reported in this publication was supported by the National Institute of Nursing Research (NINR) of the National Institutes of Health under award number 5R01NR019229, “Expanding and Scaling Two-way Texting to Reduce Unnecessary Follow-Up and Improve Adverse Event Identification Among Voluntary Medical Male Circumcision Clients in the Republic of South Africa.” The content is solely the responsibility of the authors and does not necessarily represent the official views of the National Institutes of Health."

Please state what role the funders took in the study. If the funders had no role, please state: "The funders had no role in study design, data collection and analysis, decision to publish, or preparation of the manuscript.""

If this statement is not correct you must amend it as needed. 
Please include this amended Role of Funder statement in your cover letter; we will change the online submission form on your behalf.

Response: We have amended the financial disclosure statement in the manuscript and included it within the cover letter.

5. Your ethics statement should only appear in the Methods section of your manuscript. If your ethics statement is written in any section besides the Methods, please move it to the Methods section and delete it from any other section. Please ensure that your ethics statement is included in your manuscript, as the ethics statement entered into the online submission form will not be published alongside your manuscript.

Response: The ethics statement is now in the methods section.

6. Please include captions for your Supporting Information files at the end of your manuscript, and update any in-text citations to match accordingly. Please see our Supporting Information guidelines for more information: [http://journals.plos.org/plosone/s/supporting-information.](https://urldefense.com/v3/__https:/eur01.safelinks.protection.outlook.com/?url=https*3A*2F*2Furldefense.com*2Fv3*2F__https*3A*2F*2Feur01.safelinks.protection.outlook.com*2F*3Furl*3Dhttps*3A*2F*2Furldefense.com*2Fv3*2F__http*3A*2Fjournals.plos.org*2Fplosone*2Fs*2Fsupporting-information.__*3B!!K-Hz7m0Vt54!iInB6z4ybzPWlCG2gI2YNHJIr3qlZwTOL_v1dAqDfBVTAru_5vyCkkuDN28LBQJLMQX_bujEMpJY6g*24*26data*3D05*7C01*7CGSetswe*40auruminstitute.org*7Cc639dd7a75484c4d1f4808db1c063740*7C706824277a664bae9f42f702a8ba8b2a*7C0*7C0*7C638134585501549353*7CUnknown*7CTWFpbGZsb3d8eyJWIjoiMC4wLjAwMDAiLCJQIjoiV2luMzIiLCJBTiI6Ik1haWwiLCJXVCI6Mn0*3D*7C2000*7C*7C*7C*26sdata*3DWkPyyOwbwPniLRwnGzBVIhB86pzqfNmlVtxVTVYlpfE*3D*26reserved*3D0__*3BJSUlJSUlJSUlJSUlJSUlJSUlJSUlJSUlJSUlJQ!!K-Hz7m0Vt54!igS3sJLUCpHGFDsPHZwmkI7hh1g7teQjp67FwKod5uZPLk3LnSSqek-q6KWJ4rDZk7PvNhdmMhOjiPHLtec*24&data=05*7C01*7Crmukora*40auruminstitute.org*7Cf0dc02085582472f4c6908db1df7e6b6*7C706824277a664bae9f42f702a8ba8b2a*7C0*7C0*7C638136723204775393*7CUnknown*7CTWFpbGZsb3d8eyJWIjoiMC4wLjAwMDAiLCJQIjoiV2luMzIiLCJBTiI6Ik1haWwiLCJXVCI6Mn0*3D*7C3000*7C*7C*7C&sdata=dzQ6x4nItdzKkq0IsB*2BJLnWrsXRUbId9j8F0QlcmN8U*3D&reserved=0__;JSUlJSUlJSUlJSUqKioqKioqKioqKiolJSoqKioqKioqKioqKioqKiUlKiUlJSUlJSUlJSUlJSUlJSUlJSUlJQ!!K-Hz7m0Vt54!iJ0i0UU-hjYj9FMAXIBNliosAbEuCa07IQoTA4EGsCLHhE2hyklN6QLFPRkRatB-FDTU4RES0ohpOaUMb9Y$)

Response: We have included captions for our supporting information files and updated the in-text citations to match accordingly.

**Part 2: Review Comments to the Author**

**Reviewer #1**

General Comments
The authors are to be commended for designing and completing an rigorous study comparing alternative ways to handle VMMC post-op care. While the main finding of reduced use of in-person health services associated with the two-way texting (2wT) compared to routine in-person follow-up is not in question, there is some concern about who realizes the cost-savings and the implications for the health sector. As shown in Table 4, the bulk of the cost savings from 2wT comes from reduced follow-up visits (0.22 per person in the 2wT cohort (line 254) vs. 1.34 in the routine cohort (line 252)). Yet, these differences are more likely to result in financial (transport) and time savings (opportunity costs) to the clients than cost savings to the health system. I understand your attempt to monetize these as savings to the health system by considering three hypothetical scenarios, but the fact remains that these will be savings to the client and therefore it is disingenuous to talk about these as savings to the health system as you do in the conclusion. In fact, the savings within the health system are most likely going to be labor time savings (opportunity costs) and not monetary savings due to reduced use of consumable supplies. If you remove the cost of transport for follow-up visits (~$3.91 from line 287), then there would no longer be a monetary savings to the health sector. This does not mean that this type of intervention is not worth supporting as it does not lead to worse outcomes and is certainly more convenient to the clients while at the same time reducing the workload burden within the health system.

I would encourage the authors to consider presenting the results with stratification as to who bears the cost (health system or clients) rather than create the hypothetical scenario where client costs are loaded onto the health system. This would make the analysis more transparent and avoid the trap of thinking of the savings as monetary savings when the majority are opportunity cost savings as current health care staff are now available to address other needs.

Response: We highly appreciate your comments and have expanded our explanation and support for this perspective in the paper. n South Africa, as in most PEPFAR-funded programs (which is the vast majority of VMMC programs in sub-Saharan Africa), it is the donor who funds the implementing partners who implement the VMMC programs. Costs to clients in these largely vertical programs are minimized so that uptake is high to achieve HIV prevention saturation goals in minimal time. Although this intensive funding may end, it is the current model and is likely to remain this way for the next 5 years. While some clients may pay for transport for VMMC services in urban areas where services are typically more convenient, in rural areas, it is more common that the program picks up related transport costs for both the VMMC procedure and all follow-up visits. It is also common that men with potential complications would request transport to the clinic or have a clinician make a home visit. This low-cost to client model is an incentive for the man to get circumcised (reducing barriers to VMMC) and have follow-up but is a heavy cost born by the programs. In this Aurum/CHAPS program where the RCT was conducted, this is also the model: urban clients largely are responsible for getting to their follow-up visits where rural clients have their transportation costs paid to encourage visit attendance. As with the previous Zimbabwe study, the payer perspective makes sense for this analysis as it is largely that program, the payer, that bears these follow-up costs and stands to gain the most from the 2wT approach to reduce those unnecessary visits. We agree also that the bulk of the cost savings from 2wT comes from reduced follow-up visits (0.22 per person in the 2wT cohort vs. 1.34 in the routine cohort). We also removed the three hypothetical scenarios. We hope the reviewer will agree with the expanded explanation and support for this approach.

Specific Comments:
Line 327: Where does the $7.6 (76%) figure come from. Looking at Table 4, should the denominator be $6.48 and the percentage 83%? Also, it is customary to report USD with 2 decimal places (applies throughout text and figure 1.

Response: We have updated the results and the narratives. We also updated the texts, Tables, and Figures to 2 decimal points.

Table 4, can you include “$” in the Total cost row so it is clear that these are all USD amounts?

Response: We added “$” in the tables.

Fig 1: Please add a vertical axis title making it explicit that these are USD amounts in the bars.
Fig 2: Please add a vertical axis title making it explicit that these are USD amounts in the bars.

Response: We revised figures to include $ signs.

Table 5: Label second data row as Routine – 1.34 visits (observed).
Response: Yes.

Lines 387 – 394: This is where I think you are overselling your results. These will not be monetary savings that can be reprogramed to different uses. Most will be time savings for existing staff which can be repurposed as discussed in the preceding paragraph.
Response: Yes. We have revised our statements accordingly.

Lines 414-416: Rephrase this once you know where the cost savings occur (health sector or clients).

Response: Yes. We have revised our statements accordingly.

**Reviewer #2**

This is a well-conducted costing assessment of an SMS-based alternative to routine follow-up care for VMMC compared with standard of care, which involves several return clinic visits. The intervention is relevant and well described, and the need to assess cost and potential cost-savings is also well established. I am not an expert in costing analyses and certainly suggest that at least one reviewer be selected who is such an expert. However, based on my reading it seemed to be both well conducted and clearly presented. I found the overall manuscript to be well written and appropriately justified in each section. I did see one or two potential wording edits – for example, I would suggest that all results be reported in the past tense. However, I overall believe this is a strong manuscript and support its publication.

Response: The results have now been reported in the past tense.

**Reviewer #3**

Please see my reviewer report attached separately. It will be easier to reach that document as formatted rather than writing more here.

Reviewer Report

Cost savings in male circumcision post-operative care continuum in rural and urban

South Africa: Evidence on the importance of initial counselling and daily SMS

**Summary**

1. Severe adverse events (AEs) are relatively rare after VMMC in South Africa and elsewhere. As a result, requiring men to attend multiple post-op visits likely wastes men’s time, wastes public resources, and probably adds to congestion at already crowded clinics. Rather than requiring men to return to clinics, implementing two-way text messaging (2wT) during the 14-day post-op period yielded good results in Zimbabwe, and it seems the same team implemented a similar study in South Africa (Feldacker PI). While the current costing manuscript provides a reference 15 for the main study, I could not find this reference online to review the primary results of the study, which is useful for understanding the evaluation of costs of services in each study arm.

Response:

We have updated the reference as shown below:

Feldacker C, Pienaar J, Wasunna B, Ndebele F, Khumalo C, Day S, Tweya H, Oni F, Sardini M, Adhikary B, Waweru E, Barasa M, Dixon A, Jafa K, Su Y, Sherr K, Setswe G

Expanding evidence on safety and efficiency of interactive, SMS-based telehealth for voluntary medical male circumcision follow-up as compared to in-person reviews: A randomized control trial in rural and urban South Africa

Journal of Medical Internet Research. 24/02/2023:42111 (forthcoming/in press)

2. As provided by the authors (line 87), “The objective this study was to estimate the costs associated with 2wT telehealth follow-up as compared to routine in-person VMMC follow-up in rural and urban South Africa.” And on line 162, costs were to be estimated using an “activity-based costing approach (with 4 main activities: counseling, sms follow up, physical follow up, and tracing).

Response: Correct.

**Main comments.**

3. While the objective and costing approach are both logical, my basic comment on this analysis is that costing approach presented here is not logically developed or presented. I’ll explain more below.

Response: Our approach is rigorous in conceptualization and analysis. See our responses to your following comments.

4. In principle, the evaluation of costs of services provided for post-op care for patients in each study arm requires information on the quantity of resources provided to each patient (e.g., services at clinic visits; 2wT services, etc.) and a unit cost for each service. With 537 clients in standard care and 547 in the intervention group, a data set with 1084 observations containing the list of resources included in the costing analysis along with the quantities should exist. This is often call the resource use matrix (see, e.g. <https://www.heroza.org/researchtools/the-healthcare-cost-and-outcomes-model-hcom/> ). This dataset can then also include any primary or secondary study outcomes as relevant for a study. For documentation purposes, this dataset (sometimes called a resource use matrix) should be provided as a supplemental file with this paper (or the dataset could be put in a data repository; deidentify as needed but the basic resource variables required for costing do not identify clients). This also implies that the data availability statement is not adequate. All relevant data are not provided within the manuscript.

I would highly recommend that you follow this approach for your costing analysis as explained by the HERO costing model. You can look at the Scott et al. article for an example of applying this approach: <https://bmcpublichealth.biomedcentral.com/articles/10.1186/1471-2458-14-296>

Note: This study has separate unit costs for each site in the study (for some resources).

A similar type of analysis for clinical trial is used in this study:

<https://www.thelancet.com/journals/langlo/article/PIIS2214-109X(21)00039-5/fulltext>

Look at supplemental appendix 2 for costing details.

One you revise your analysis and rewrite your manuscript (basically a new paper), you will then have a logically presented cost analysis for your topic.

Response: Thank you for recommending the HERO costing approach, with several unique and interesting applications that we are considering in future papers. However, for this paper, we are remaining consistent with our previously published approach and in line with PEPFAR costing considerations. For this paper, we are confident about our current costing model – now revised considerably with your inputs and suggestions. We’ll consider HERO in future studies.

**The comments below are just some extra comments or information related to how you can revise your analysis and presentation.**

5. As noted on line 164, there were four activity categories in costing: counselling on the day of male circumcision, SMS follow ups, physical follow-up visits, and tracing. With activity-based costing, I’d expected to see a description of how a unit cost for each activity would be created (and then combined with the resource use matrix) to estimate a cost for each activity and then a total cost of post-op follow up for each patients (the set of unit costs can also be provided with the resource use matrix). With this information, standard summary statistics can be provided (mean costs per client by study arm, median/IQR, etc.) and then you can estimate the mean difference between study arms (with 95% CI, etc.).

Response: We specify data input for costing results, unit cost for each activity and sub categories, and total cost for each patient in the excel spreadsheets (Cost Analysis Tool, xlsx). We share it as an appendix and the readers can use it to further change the parameters for further costing analysis.

6. I’m not sure the cost of AE treatment should be considered a cost for this analysis. In Zimbabwe, the 2wT increased slightly the identification of AEs (as the authors note due “improved AE ascertainment”). If 2wT serves as a proxy for active surveillance, identifying AEs is actually a benefit of the intervention. <https://journals.lww.com/jaids/Fulltext/2020/01010/Reducing_Provider_Workload_While_Preserving.3.aspx>). It seems this is basically a type of information bias, in that AEs are likely identified better in one study arm.

Response: The goal of the program is to ensure safety and quality service for post VMMC patients. It is unethical to leave AE treatment out.

7. Line 94 – since you are estimating costs based on a two-arm individually randomized clinical trials, I suggest just using Study Arms or Study Groups

Response: Yes. We use study arms as you recommended.

8. Line 139 – How were data collection tools piloted? These tools/files can also be provided as a supplemental file or with the data repository mentioned above.

Response: We have included ‘S2 Appendix Time and Motion Form’ as a supplemental file.

9. Line 148 – Line 149: While not a big deal, it would help to be more specific on the assumptions used to estimate a nurse wage per hour (I assume used with time information to get a time cost for various services). First, regarding wage per hour, the authors need to confirm that they obtained the total cost to employer. In addition, I assume nurses have days for holidays, annual leave, perhaps other types. These total days off need to be subtracted from some maximum possible to come up with a wage per hour working.

Response: Yes, we obtained the total cost to employer, including salary and fringe. The standard workweek in South Africa is 5 days per week, from Monday to Friday. We use this standard in calculating hourly wages and specified it in the Methods section. We acknowledge in the Discussion section that some nurses work on weekends and public holidays, which would increase the number of working hours. In addition, some nurses may have additional days off, such as annual leave or sick leave, which would decrease the number of working hours.

10. Line 154-160 – These hypotheses are rather odd for this costing paper. For example, this costing paper is designed to estimate the cost of post-op care by study arm. Once the costs by study are presented (e.g. mean(SD), median(IQR)), it is then possible to estimate a mean difference with a 95% CI. The second topic is simply the same analysis stratified by urban rural (so provide the same results stratified by urban/rural). I’m not sure why Hyp 3 is included. As presented in Table 1, the 2wT arm does not require post-op visits.

Response: We removed the 3^rd^ hypothesis. The first hypothesis is the primary motivation for the costing analysis. The second topic is stratified by location as we are interested in understanding rural/urban disparities.

11. Line 165 – Table 2 provides some sort of summary results for various stuff, but I would not consider them “parameters”. I have no idea why you mention 50%+- uncertainty intervals here either.

Response: Yes, these are summary statistics and we changed the table title accordingly. We used uncertainty intervals in the same approach as the published paper about 2wT costing in Zimbabwe for consistency.

12. Line 181 – I now see what the authors are trying to do. I’ll break out the SMS cost in detail as described in the paragraph above line 181.

SMS cost =	set-up cost +

SMS unit cost * average number SMS per client +

(1/60)*nursewage*average number of SMS per client

I think it would be much better to use the actual patient level data along with the unit cost to first estimate the SMS cost per patient. With 500+ observations in each study arm (but SMS only in one arm), you have 500+ SMS cost numbers (which later you can summarize, etc.).

The resource use matrix would have the follow quantity resources:

X1 = Set up SMS for client (0 for standard care, 1 for 2wT arm)

X2 = Number of SMS to client (0 for standard care, looks like 13 if exactly one each day, but like more or less for some clients).

A unit cost for each resource is then:

C1 = cost to set up SMS

C2 = cost per SMS = (SMS unit cost + (1/60)*wage)

So for each client, SMS cost = C1*X1 + C2*X2

In Line 174, this same process can be used for counseling time, unless you do not have counselling time captured per client. It’s OK if you just have a counseling time by study arm (seems like 2wT arm has longer post counseling).

Response: Thank you for detailing this alternative approach. We appreciate your advice. The results would be the same by the end of the costing analysis, either using individual-level data first and summarize as the next step or using group mean in costing.

13. Line 191 – Here again, you can have the number of rural visits and number of urban visits per patient from your data, and then it looks like you have one “unit cost” for a rural visit.

Response: Yes. We had a dimensionality reduction in costing. We used the average visits in costing. The results would be the same by either approach.

14. I suspect you have an Excel file that includes all the calculations you used to estimate a transport fuel cost and transport time cost, etc. It would be good to add that file to the study data repository.

Response: Yes. We add the file to study data repository. Good advice.

15. In line 194, do you have a fuel cost per client? Or are these based on some assumed averages? If you do, then you can have a fuel cost per client per visit that is specific to each client. In this case, you’d have km roundtrip to client as a quantity in the resource use matrix. For each client, the number of rural trips and urban trips would be extra quantity variables in the resource use matrix. Petrol price would then be an extra unit cost to add into the analysis and fuel economy would then be an extra parameter used to estimate the fuel cost per client (and similar logic for transport time, review time, etc.

Response: We had fuel cost from provider time-motion study. We used average fuel cost in our analysis in rural areas. We didn’t include client transportation costs in our analysis as we took the payer perspective instead of patient or societal perspective in costing.

16. It seems some asset value per trip (for the vehicle) is needed that included the asset value plus other costs (annual road taxes, license, maintenance, insurance, etc.)?

Response: This study focuses on cost of the activities to serve an average client, given that 2wT is implemented within an existing routine VMMC service. Accordingly, start-up costs (e.g., developing text message library, translation to local languages, adaptation to South Africa context) and fixed costs (e.g., full-time employment dedicated to 2wT, cell phones, computers, vehicle purchase, insurance, maintenance cost) were excluded.

17. Line 203 -- for phone tracing, you have the number of tracing attempts per client, so use that directly (e.g., # unsuccessful attempts over follow up period, # successful). Then you have a unit cost for each type of event.

Response: We didn’t specify unit cost for each type of event (i.e., successful, or unsuccessful) as we are interested in the phone tracing costs in lump sum.

18. Line 211 – same issue here for tracing.

Response: We didn’t specify unit cost for each type of event (i.e., successful, or unsuccessful) as we are interested in the home tracing costs in lump sum.

19. Line 221 – It is not clear what the authors did for the analysis presented in Table 3.

Response: We removed Table 3 as you and another reviewer suggested.

20. Line 225 – The purpose of Table 2 and the information presented in Table 2 is unclear. You have two study arms. I highly recommend you follow your earlier stated objective (estimate costs in each study arm using activity-based costing and I’d add the actual patient level data that you have.

Actually Table 4 comes close to what you want to report. Once you do the client level cost analysis, you can present the resource quantities per client, the unit costs that go with each resource, and the costs by resource. You can report for all means(SD) and median (IQR),, etc, and then estimate the mean differences (with 95% CI). A separate table can then report the same information stratified by urban/rural. By the way, is this urban/rural clients or where the clinics are located?

Response: The purpose of Table 2 is to present summary statistics that were relevant to routine and 2wT costing. We have simplified Table 2 to make it more digestible. We also revised Table 4 to present rural, urban, and averages. There were urban/rural clients in our stratification.

21. I’m not going to add a lot of additional comments on the rest of the paper (e.g., issue in line 317 – Table shows cost of post op care, not per visit I think), given that the methods and presentation needs to be fully revised. With the revised paper, the section on “other scenarios” doesn’t really make sense. Check line 428

Response: We made major revisions in our methods, result presentation, and narratives. Thank you again for all your valuable feedback. We appreciate your review.
