# Supplementary material for: Cost savings in male circumcision post-operative care using two-way text-based follow-up in rural and urban South Africa
Source: PLoS One. 2023 Nov 16;18(11):e0294449. doi: 10.1371/journal.pone.0294449 (PMC10653449; doi:10.1371/journal.pone.0294449)
Supplement: Attachment — Submitted filename: Response to Reviewers 9.29.23.docx [file pone.0294449.s005.docx]

**Response to reviewers**

PONE-D-23-01844R1

Cost savings in male circumcision post-operative care using two-way texting mHealth in rural and urban South Africa

**September 29, 2023**

*Dear Plos ONE Editorial Team,*

*Thank you very much for the opportunity to revise the manuscript again to comply with these additional helpful comments. We have reviewed and updated the text, responded to the remaining suggestions, and provided an explanation for outstanding issues which we hope will alleviate any remaining issues before consideration of publication. We respond in italics to the remaining issues and provided a track changes version to review additional revisions, edits, and updates for clarity throughout.*

Editor: I do agree with the first reviewer that the results of the study are not being adequately contextualized and are even being "oversold" as to their importance. The manuscript does hold merit if the authors are able to make some few remaining adjustment. Specifically, the discussion section begins with a statement regarding the average cost-savings per client in this study. Two very important points are not well addressed.

First, nearly all the costs savings were seen in the rural intervention setting. In fact, the 2wT intervention was more costly than routine follow-up in the urban setting. Yet, your conclusion begins "With evidence from two RCTs in Zimbabwe and South Africa demonstrating 2wT safety and cost benefits in urban and rural contexts, the health sector should invest in 2wT." Results from this study do not demonstrate a cost benefit in urban settings, and recommendations to invest in the intervention would only benefit programs with a larger rural client population, based on costs alone.  Your estimates of the potential cost benefits are predicated on your findings that included a sample with equal numbers of participants in both the urban and rural settings for both study arms. This condition needs to be made clear. Second, as one reviewer pointed out, the majority of savings are from follow-up visits and driven predominately by cost-savings in provider time (Table 3). As the first reviewer noted, "These will not be monetary savings that can be reprogramed to different uses. Most will be time savings for existing staff which can be repurposed..."  This also needs to come out more clearly in the text. Both of these points will likely be key considerations for a country or a program that might consider this intervention.

Response:

*Thank you for this opportunity to provide clarity on why the rural savings would lead to overall savings in the program. Under the Aurum and CHAPS program, VMMC services are delivered predominantly in rural areas, up to 90% of the patients are from rural settings in any month as published in our clinical JMIR paper. We now added this statement in several areas of the paper, including in paragraph 5 of the introduction, “The VMMC program in SA is predominantly implemented in more rural areas [14].” We also enhanced our discussion of the savings in rural areas and explained why we think that the minimal additional costs in urban areas are a worthy investment.*

*We also softened and updated the language of the discussion about the cost savings, “2wT has additional benefits for cost savings that could lead to potential long-term cost-savings in post-VMMC care delivery. Firstly, enhanced counselling incurred a minimal cost of $0.63, but enabled the clients to effectively identify and communicate AE concerns in daily SMS – a worthy investment worthwhile cost. Second, daily SMS communication improved early detection of AEs and subsequent swift referral of those in need for in-person clinical visits. This triaging process led to identification of AEs earlier with less severity, likely averting costs of more severe AEs[14]. Third, only the clients with AE concerns or those desiring reassurance attended clinic visits, resulting in only 0.22 elected visits per 2wT client (Table 3), a reduction in review workload. Lastly, reduced follow-up visits allow providers to concentrate on cases in need of medical attention and free up time to commence surgery early and on-time, potentially increasing client satisfaction.”*

Lastly, one smaller point noted by review 1 is that both figures are missing axis titles. Please add so the figures are able to stand alone and be completely understood.

Response:

*Completed.*

Reviewer #1: General Comments:

Thank you for the clarification that the PEPFAR supported programs are covering the transport costs for clients in the rural area. Also, the emphasis on looking at costs from the payer’s perspective. However, I am still concerned that while the PEPFAR-funded program (CHAPS) will see savings (monetary cost) in reduced transportation fuel costs that the reduced staff time required for follow-up (an opportunity cost not a monetary cost savings) means that overall monetary costs to the program are likely to increase.

*Response:*

*These will be monetary savings that can be reprogramed to different uses, as we noted in the paper, like time savings for existing staff who would not be completed unnecessary reviews. In contrast, opportunity cost refers to the value of the next best alternative that one foregoes when making a decision, choosing one option always comes at the cost of not choosing another. In our study, we estimated time costs for health workers and monetize time costs by using the salary information. We didn’t estimate time savings for patients – a separate study that we did not conduct in large part as both services (VMMC) and transportation are largely provided by the program. Patient costs have been covered previously in the literature. Moreover, we look at the payer perspective since, as the reviewer notes, the provider time is being assigned to transportation – which is a wasted resource. While we agree, and we thank the reviewer, for these concerns and suggestions, it is not for the research team to interfere in the delivery of routine services nor prescribe how routine HCWs are assigned duties or duty stations. We also hope that others will view that information with an eye for applying 2wT to those settings or investigating other more efficient pathways to provide follow-up care. Using 2wT would, indeed, reduce a lot of clinician time that appears wasted in transportation time in rural areas, especially.*

I am still troubled by the conclusion that the use of 2wT could yield savings of over $1.1 million per year (line 442). As stated above, the labor savings are not monetary savings so there is no budgetary reduction associated with these “savings”. Also, I’m not sure there is evidence to suggest men’s hesitancy to take up VMMC because of the need to return for follow-up visits and with more efficient follow-up that uptake would increase (lines 444-446).

*Response:*

*Transportation and associated costs for VMMC follow-up visits are a burden to men in SA, with much literature to suggest that many men try to avoid follow up visits (from migration, employment obligations, stigma, able to heal independently) and that there is overreporting of follow-up. We added 2 additional references [10, 11] to the introduction in support of that statement. Not requiring visits but providing this telehealth opportunity to interact, appears to be a win-win for men who receive support to heal well and their nurses who can provide this care without traveling.*

*We rephrased the conclusion to remove the $1.1 million per year, to read, “Evidence from two RCTs in Zimbabwe and South Africa demonstrate that this 2wT approach provides high-quality VMMC follow-up as compared to required in-person reviews and lowers overall VMMC program costs. Rural savings using 2wT offset nominal increased costs in urban areas. Additional 2wT associated improvements in care quality, supervision, and verification also likely leading to longer-term savings. The health sector should invest in 2wT. In the context of National VMMC targets of 350,000 to 500,000 VMMCs per annum, employing 2wT could dramatically reduce the number of in-person post-operative reviews, resulting in concrete efficiency gains and significant annual program savings. Invested these resources back into the VMMC program could further expand VMMC access, improve care quality, and advance VMMC program goals of safe, efficient, and effective VMMC scale-up.”*

Specific Comments:

Line 214 & 215 (& perhaps elsewhere) replace “Aes” with “AEs”. My guess is your word processing software was trying to be helpful .

*Response: Completed.*

Line 242-244: The decision to only count the cost for a successful attempt even if multiple attempts were required seems to put downward bias on tracing costs. Please justify this decision.

*Response:*

*Documentation of attempts at tracing were poor. Therefore, we only have anecdotal support on previous attempts. This is the case and further supports the idea that 2wT could actually offset additional costs that were underestimated in this costing approach.*

Line 360: According to Line 113, NDoH protocols already require two post-surgery follow-up visits. So why is this being treated as a hypothetical scenario?

Response:

*Adherence to these guidelines varies widely, but the guidelines remain unchanged. With COVID19, there is also growing acceptance that telehealth or telephone follow-up may be acceptable. Although there are formal policies requiring 2 post-operative visits, we provided evidence that telehealth may be acceptable to replace in-person reviews for low risk men according to new PEPFAR documents about follow-up for low-risk men, but Low risk” has not been defined. The NDoH, PEPFAR, and the VMMC community need this evidence to further advocate for policy change – which is often slower than practice moves.*
